# Joel Habener, Svetlana Mojsov, and Lotte Bjerre Knudsen awarded Lasker prize for pioneering work on GLP-1

**DOI:** 10.1172/JCI186225

**Published:** 2024-09-19

**Authors:** Hossein Ardehali

**Affiliations:** Feinberg Cardiovascular and Renal Research Institute, Northwestern University school of Medicine, Chicago, Illinois, USA.

The Lasker Foundation has awarded the 2024 Lasker~DeBakey Clinical Medical Research Award to Joel Habener (Massachusetts General Hospital), Svetlana Mojsov (Rockefeller University), and Lotte Bjerre Knudsen (Novo Nordisk) for their pioneering work on glucagon-like peptide 1 (GLP-1) receptor agonists and their applications to obesity. This recognition highlights the revolutionary work of these scientists that led to the discovery of one of the most exciting family of drugs in our lifetime.

## The road to discovering GLP-1

**Figure 1:**
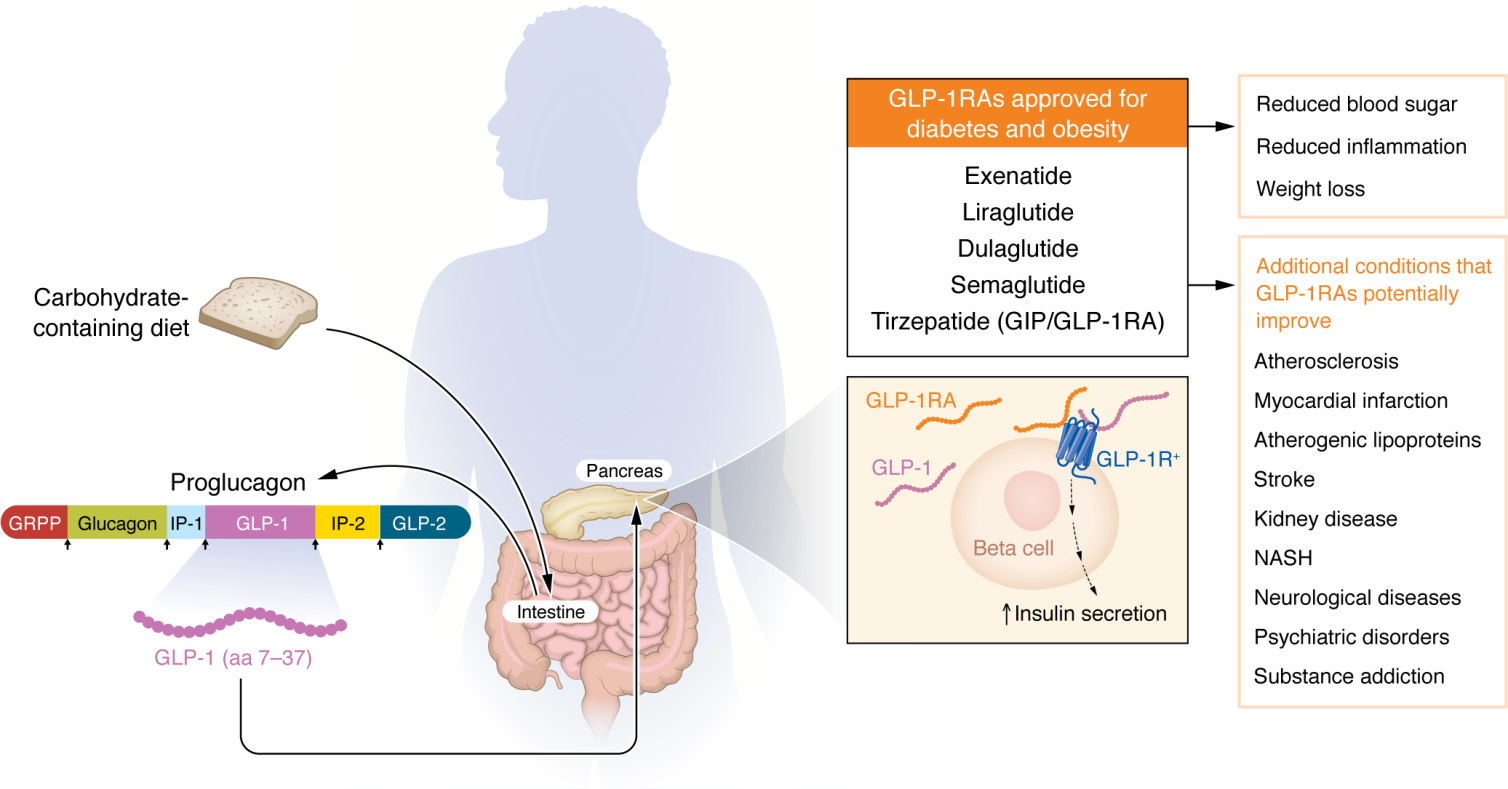
Mechanism of GLP-1 production and its beneficial effects. Proglucagon is released from intestinal cells and is processed into its components, one of which is GLP-1. GLP-1 binds to its receptor in pancreas and leads to insulin secretion. GLP-1 exerts additional beneficial effects by binding to the GLP-1 receptor in other tissues or via indirectly reducing inflammation. The list of FDA-approved GLP-1RA drugs is also included in the figure.

In the early 1980s, Joel Habener and his team isolated cDNA encoding proglucagon using a library generated from anglerfish pancreatic islet cells (1). This discovery was later followed by the identification of the rat proglucagon cDNA sequences (2, 3). Around the same time, Graeme Bell at the University of Chicago identified the proglucagon cDNA and genes from hamsters, cows, and humans (4). Both mammalian and fish sequences revealed that, in addition to glucagon, the cDNA also contained glucagon-related sequences, which resembled another gut-derived hormone that had been discovered in 1970s, i.e., the incretin glucose-dependent insulinotropic polypeptide (GIP). Bell’s work showed that mammalian proglucagon contained two GLP regions, which were termed GLP-1 and GLP-2 (Figure 1), with GLP-1 highly conserved among mammals (4). These earlier studies demonstrated that glucagon and GLPs, similar to insulin and parathyroid hormone, are generated as part of a larger prohormone and are posttranslationally cleaved to produce the final peptides. Later, specific endopeptidases called prohormone convertases were discovered to cleave the prohormones at basic sites containing the amino acids arginine and lysine.

In the summer of 1984, Daniel Drucker joined the Habener lab as a postdoctoral fellow and conducted seminal studies characterizing the predicted proglucagon products and their bioactivity. He transfected a proglucagon cDNA into islet cells, pituitary cells, and fibroblasts. While there was minimal processing in fibroblasts, he was able to show that the bioactive form of GLP-1 was amino-truncated at a single basic site and that both GLP-1 and GLP-2 were processed within transfected pituitary and islet cells (5). After establishing his own lab in Toronto, Drucker continued to unravel the physiologic action of GLP-1 and GLP-2 and the molecular control of proglucagon expression and processing. He showed that intestinal proglucagon cDNA is identical to the form expressed in pancreas (6). However, further studies also showed that, in contrast to the pancreas, immunoreactive GLP-1 peptides in the intestinal extracts consisted of only smaller peptides, indicating differential processing in the gut and greater efficiency in processing proglucagon into smaller peptides compared with the pancreas (6). This finding is consistent with the incretin nature of these peptides in that oral nutrients stimulate their production in the gut and not in the pancreas (Figure 1).

Drucker also identified GLP-2 as an intestinal growth factor since its administration was associated with protection of gut mucosal cells against injury (7). GLP-2 was also shown to increase nutrient absorption. As a result, a degradation-resistant GLP-2 analog (developed in Drucker’s lab and later designated as teduglutide) was approved by the FDA in 2012 for chronic therapy of patients with parenteral nutrition-dependent short-bowel syndrome (8, 9).

While these studies were ongoing, Jens Holst worked on signals that regulate insulin secretion in postprandial reactive hypoglycemia after gastric surgery in Denmark. His research group focused on intestinal cells that produce glucagon, and his laboratory’s work led to the discovery of prohormone molecules glicentin and oxyntomodulin (10). These molecules contain the full glucagon amino acids, but unlike proglucagon, lack the GLP sequences. Thus, Holst and colleagues turned their focus to developing immunoassays and showed that proglucagon is cleaved in the gut (but not in the pancreas) to generate two GLPs (Figure 1). They also showed that a truncated form of proglucagon (aa 78–108) isolated from the gut can induce insulin production (11). In the 1990s, his group also showed that infusion of the same truncated form of proglucagon causes insulin secretin and inhibits glucagon production in humans (12). Further work from Holst’s group and others showed that GLP-1 can inhibit gastric motility and gastric and pancreatic exocrine function and inhibit appetite and food intake (13). These latter studies ultimately led to the use of GLP-1 agonists for treatment of obesity.

## Discovery of the bioactive form of GLP-1

While Habener’s lab was working on proglucagon, Svetlana Mojsov was independently focusing on chemical identification of the bioactive form of GLP-1. Mojsov was born in Serbia (what was called Yugoslavia at the time) and conducted her graduate studies on chemical synthesis of glucagon in the lab of the Nobel Laureate Bruce Merrifield. She then moved to Massachusetts General Hospital, and although she was at the same institution as Joel Habener, she conducted her work on GLP-1 independently. Mojsov worked to identify the biologically active form of GLP-1 by taking an alternative and complementary approach that built on her background in chemistry. Studies had shown that in rat insulinoma cells GLP-1 (aa 1–37), which was the predicted GLP-1 sequence, did not increase cAMP production. Based on her previous work on glucagon, she hypothesized that amino acids 7–37 may comprise the active form of the long-sought incretin GLP-1. She synthesized unique regions of GLP-1, and subsequently she generated rabbit antibodies to these regions of GLP-1.

Around this time, she started collaborating with Drucker and Habener and identified GLP-1 (aa 7–37) in rat intestines using the antibodies that she had developed; however, their critical experimentation was to show that GLP-1 (aa 7–37) is biologically active. Their collaborative work led to the discovery that GLP-1 (aa 7–37) peptide could stimulate insulin release in rat pancreatic islet cells (14). Additionally, in collaboration with Gordon Weir, the group demonstrated the ability of GLP-1 (aa 7–27) to increase insulin production in an ex vivo model of rat pancreas. This seminal work was published in *Journal of Clinical Investigation* in 1987 (15).

## Initial studies on the application of GLP-1 to humans

In 1992, the first study on GLP-1 in humans was conducted, demonstrating that infusion of GLP-1 (aa 7–37) peptide induced insulin section and had antidiabetic effects in both patients with and without diabetes (16). One year later, Nauck and colleagues showed that infusion of the GLP-1 peptide normalized blood glucose in patients with type 2 diabetes (17). However, subsequent studies with s.c. administration of GLP-1 were disappointing due to rapid degradation of GLP-1 by the enzyme dipeptidyl-peptidase-4 (DDP4). Inhibitors of DDP4 reduce GLP-1 degradation and cause a significant increase in circulating insulin levels. DDP4 inhibitors, also known as gliptins, such as sitagliptin, saxagliptin, linagliptin, and alogliptin, were subsequently approved by the FDA for treatment of type 2 diabetes in adults in the mid-2000s.

Holst’s group also conducted parallel studies in humans and showed that infusion of the synthetic GLP-1 (aa 7–36) amide s.c. by a pump improved plasma glucose, insulin sensitivity, HgA1C, and body weight with minimal side effects (18). This pioneering human investigation provided proof of concept for the therapeutic potential of GLP-1 and led to further effort to develop GLP-1 agonists for clinical use.

## GLP-1 receptor agonists

Despite clinical studies showing that insulin production can be stimulated with GLP-1, its short half-life (1.5 minute and 1.5 hours following intravenous and s.c. administration, respectively) made its clinical application limited. The first GLP-1 receptor agonist (GLP-1RA) approved for clinical use was exenatide, a DPP4 protease-resistant form of the hormone, which was based on exendin-4 and originally isolated from the lizard venom (19). Exenatide was initially given twice daily and was approved in 2005. Another form of exenatide that was given once weekly (under the brand name of Byetta or Bydureon) was later approved for the treatment of type 2 diabetes.

To circumvent the limitation associated with the short half-life of these drugs, scientists at Novo Nordisk, including Lotte Bjerre Knudsen, focused on developing more stable GLP-1RAs. Knudsen started working at Novo Nordisk when she was a chemical engineering student at the Technical University of Denmark. After completing her studies, she joined Novo Nordisk and focused specifically on albumin binding to increase the half-life of GLP-1RA. Albumin binds a number of insoluble substrates, including fatty acids and steroids, and facilitates their solubility and transport in blood. Knudsen’s group made a fatty acid derivative of GLP-1 to generate analogs that can bind to albumin in a reversible manner and would protect GLP-1 from degradation by DPP4 and renal filtration. These studies led to the discovery of liraglutide as the first GLP-1–based analog suitable for once-a-day dosing (20).

Given that GLP-1RAs are applied by injection, longer acting forms are desirable. The first form of long-acting GLP-1RA was taspoglutide, which showed promising results in a phase III clinical trial but was not submitted to the FDA for approval owing to rare instances of anaphylaxis. Knudsen’s group at Novo Nordisk continued to focus on albumin binding, and by modifying GLP-1 and the fatty acid moiety linker, they successfully generated semaglutide as a long-acting GLP-1RA that can be administered with a once-weekly dosage (20).

## Other beneficial effects of GLP-1R agonists

Exciting recent studies indicate that beneficial effects of GLP-1RA go beyond diabetes and obesity (Figure 1). GLP-1RAs have been studied in at least eight randomized clinical trials that assessed cardiovascular (CV) outcome in patients with diabetes (21). Four of these clinical trials met the primary endpoint and showed a significant reduction in CV outcome. Of note, semaglutide, which has the biggest market share of GLP-1RAs, was studied in SUSTAIN-6, where it achieved 26% reduction in the primary composite outcome (the first occurrence of CV-related death), nonfatal myocardial infarction, or nonfatal stroke in SUSTAIN-6 study (22). Another study, the SELECT trial, showed that, in patients with obesity without diabetes, semaglutide treatment was associated with a reduction in the incidence of CV death, nonfatal myocardial infarction, or nonfatal stroke (23). Finally, STEP-HFpEF demonstrated that treatment with semaglutide in patients with heart failure with preserved ejection fraction is associated with a reduction in symptoms and physical limitations, greater improvements in exercise function, and greater weight loss (24).

These trials collectively suggest that treatment with GLP-1RAs may exert protective effects in the heart against injury. The mechanism for this protective effect is likely multifaceted. Of course, the reduction in body weight and control of blood glucose through the antidiabetic effects of these drugs may be a key element in their cardioprotective effects. However, preclinical and clinical studies indicate that the beneficial effects of these drugs on the heart go beyond their effects on body weight and diabetes. For example, in the SELECT trial, the beneficial effects of semaglutide on the heart were higher than the degree of weight loss (23). Additionally, GLP-1RAs have also been shown to reduce blood pressure and atherogenic lipoproteins. Finally, there is now evidence that treatment with GLP-1RA is associated with a reduction in systemic inflammation, which appears to be independent of the degree of weight loss and could also explain the CV beneficial effects (25).

GLP-1-RAs are also studied in liver disease and kidney disease. The FLOW trial showed that semaglutide reduces the risk of clinically important kidney outcomes and death from CV disease in patients with diabetes and chronic kidney disease (26). The ESSENCE study is a phase III clinical trial currently studying the effects of semaglutide in metabolic dysfunction-associated steatohepatitis. The mechanism for the protective effects of GLP-1RA in the liver and kidney are not clear, but the effects could be through other cells that express GLP-1R within these organs. The antiinflammatory effects of these drugs could also contribute to their beneficial effects (Figure 1).

The effects of GLP-1RA in neurological disorders are also being studied. Two phase III clinical trials, EVOKE and EVOKE Plus, will study the effects of oral semaglutide (which is currently not FDA approved) in patients with cognitive deterioration (27). It is also proposed that GLP-1RA may reduce addictive and psychiatric disorders. However, in the SELECT trial, there was no difference in psychiatric disorders. Cohort and electronic health record studies suggest that semaglutide may be associated with reduced incidence of suicidal ideation (23). Finally, post hoc analysis of some studies suggests that GLP-1RA may be associated with a reduction in alcohol intake (27).

Despite their success in reversing a number of major disorders, the use of these drugs is associated with a number of side effects, including nausea, vomiting, pancreatitis, and injection site reactions (28). Some of these side effects appear to be due to the binding of these drugs with GLP-1R in the brain. Another major side effect of these drugs is muscle loss, which can be as high as 40% in some patients and may be worse in older patients. There is also a concern that if the drug is discontinued, the weight gain would be totally in the form of fat, leading to a net muscle loss in those patients (29).

## Concluding remarks

The discovery of GLP-1RAs was pioneered by several investigators and has led to a new treatment strategy for diabetes and weight loss. These drugs have also been shown to have beneficial effects on other organs that go beyond their metabolic effects, making them potentially applicable for the treatment of additional disorders. GLP-1RAs have revolutionized the treatment for metabolic disorders, and the pioneers in this field truly deserve the recognition for their groundbreaking discovery.

## Version 1 09/19/2024

Electronic publication

## Version 2 09/20/2024

Corrected typographical error

## Version 3 09/26/2024

Electronic publication
